# Sex differences in mouse Transient Receptor Potential Cation Channel, Subfamily M, Member 8 expressing trigeminal ganglion neurons

**DOI:** 10.1371/journal.pone.0176753

**Published:** 2017-05-04

**Authors:** Robert M. Caudle, Stephanie L. Caudle, Alan C. Jenkins, Andrew H. Ahn, John K. Neubert

**Affiliations:** 1 Department of Oral and Maxillofacial Surgery, University of Florida College of Dentistry, Gainesville, Florida, United States of America; 2 Department of Neuroscience, University of Florida College of Medicine, Gainesville, Florida, United States of America; 3 Department of Orthodontics, University of Florida College of Dentistry, Gainesville, Florida, United States of America; 4 Department of Neurology, University of Florida College of Medicine, Gainesville, Florida, United States of America; National Eye Centre, UNITED STATES

## Abstract

The detection of cool temperatures is thought to be mediated by primary afferent neurons that express the cool temperature sensing protein Transient Receptor Potential Cation Channel, Subfamily M, Member 8 (TRPM8). Using mice, this study tested the hypothesis that sex differences in sensitivity to cool temperatures were mediated by differences in neurons that express TRPM8. Ion currents from TRPM8 expressing trigeminal ganglion (TRG) neurons in females demonstrated larger hyperpolarization-activated cyclic nucleotide-gated currents (I_h_) than male neurons at both 30° and 18°C. Additionally, female neurons’ voltage gated potassium currents (I_k_) were suppressed by cooling, whereas male I_k_ was not significantly affected. At the holding potential tested (-60mV) TRPM8 currents were not visibly activated in either sex by cooling. Modeling the effect of I_h_ and I_k_ on membrane potentials demonstrated that at 30° the membrane potential in both sexes is unstable. At 18°, female TRPM8 TRG neurons develop a large oscillating pattern in their membrane potential, whereas male neurons become highly stable. These findings suggest that the differences in I_h_ and I_k_ in the TRPM8 TRG neurons of male and female mice likely leads to greater sensitivity of female mice to the cool temperature. This hypothesis was confirmed in an operant reward/conflict assay. Female mice contacted an 18°C surface for approximately half the time that males contacted the cool surface. At 33° and 10°C male and female mice contacted the stimulus for similar amounts of time. These data suggest that sex differences in the functioning of I_h_ and I_k_ in TRPM8 expressing primary afferent neurons leads to differences in cool temperature sensitivity.

## Introduction

In designing comfortable work spaces environmental engineers often encounter difficulty in establishing the appropriate room temperature for the employees occupying that space when they are of mixed gender. Research into this problem has established that females generally find cool temperatures (18°C to 22°C) very uncomfortable, whereas male workers find these temperatures acceptable. The higher temperatures that females find comfortable males often report as intolerably warm [1]. This divergence in comfort likely arises from differences in the neurophysiology of the sensory systems of males and females. These sensory differences also trigger more robust autonomic responses in females to cool temperatures [2], which may enhance the discomfort by reducing blood flow to the extremities.

For more than a decade it has been established that primary afferent neurons that express the temperature sensitive protein Transient Receptor Potential Cation Channel, Subfamily M, Member 8 (TRPM8) are responsible for detecting skin surface temperatures that drop below 22°C. TRPM8 is a non-selective cation channel that is activated at these temperatures [3–8]. Therefore, the current study tested the hypothesis that differences in male and female sensitivity to cool temperatures may be the result of differences in the expression or function of TRPM8 in their primary afferent neurons. To test this hypothesis, we examined TRPM8 expressing neurons from the trigeminal ganglia of male and female mice and compared their ionic channel profiles with the behavioral responses of animals in an operant orofacial thermal nociception assay. These data led to the conclusion that differences in cool temperature sensitivity in male and female mice are the result of divergent ion channel responses to cool temperatures in the TRPM8 expressing neurons.

## Methods

### Mice

TRPM8^tm1Apat^/J knockout mice (Jackson Labs, Bar Harbor, ME) were crossed in house with C57BL/6 mice (Charles Rivers, Wilmington, MA) to produce heterozygotes (TRPM8^EGFP-/+^,22 male and 29 female). The TRPM8^tm1Apat^/J knockout mice express enhanced green fluorescent protein (EGFP) in place of TRPM8. The heterozygotes were utilized for histological analysis of TRPM8 expressing neurons and electrophysiology. Hairless SKH1 mice (20 – 35g, 10 male and 10 female, Charles Rivers, Wilmington, MA) were utilized for behavioral experiments. Animals were housed with a 12-h light–dark cycle, and food and water were available *ad libitum*. Experiments were conducted between 09.00 and 18.00 h at a room temperature of 22°C. All experiments were approved by the University of Florida Institutional Animal Care and Use Committee and were performed in compliance with the National Institutes of Health guidelines.

### Histology

TRPM8^EGFP-/+^ mice were euthanized by isoflurane inhalation (5% in O_2_) and perfused intracardially with phosphate-buffered saline (PBS, pH 7.4) and subsequently 4% paraformaldehyde. The brainstem, trigeminal ganglia and skin from the face were removed and were fixed for 15 h in 4% paraformaldehyde, and then transferred to 30% sucrose in PBS for 24–30 h. The tissues were frozen and transverse sections cut at 40 μm. Every third section was collected for staining. Free-floating sections were incubated in PBS, 0.3% Triton X-100 and 5% normal goat serum (NGS–T) for 1 h before incubation in primary anti-green fluorescent protein antibody at a dilution of 1: 50,000 in the NGS-T for 18 h at room temperature. Preparations were then rinsed in NGS-T 3 times for 10 min each. The secondary antibody, Alexa Fluor 594 goat anti-rabbit IgG (Invitrogen), was diluted to 1:500 with NGS-T and the preparations incubated at room temperature for 1 h. The preparations were then rinsed 3 times for 10 min each, mounted, cover slipped, and imaged.

### Electrophysiology

Male and female TRPM8^EGFP-/+^ mice were euthanized by isoflurane inhalation (5% in O_2_) and the trigeminal ganglia were removed. The ganglia were then incubated for 2 hours at 37° in Tyrode’s buffer (mM: 140 NaCl, 4 KCl, 2 MgCl_2_, 2 CaCl_2_, 10 glucose, and 10 HEPES, adjusted to pH 7.4 with NaOH) containing collagenase (Sigma, St. Louis, MO)(2mg/ml). The ganglia were triturated with a plastic pipette, pelleted by centrifugation (100 X g), resuspended with fresh Tyrode’s buffer, and plated onto 30mm poly-d-lysine coated polystyrene plates. The cells were allowed to adhere to the plates for 1 hour at room temperature prior to initiating the experiments [9–11]. During the experiments the cells were superfused with Tyrode’s buffer at 5mls per minute. The bath’s volume was maintained at approximately 1ml. The TRPM8 expressing neurons were identified by the expression of EGFP using an inverted microscope equipped with fluorescence optics (Olympus IX70). EGFP labeled cells were whole cell patch clamped with 1.5mm glass electrodes filled with electrode buffer (mM: 140 KCl, 1 CaCl_2_, 10 EGTA, 10 HEPES, 2 MgCl_2_). The pH was adjusted to 7.4 with KOH. The electrodes were pulled to resistances of 2–4 MΩ with a P-87 Flaming/Brown microelectrode puller (Sutter Instruments, Navato, CA). Data were collected using an Axopatch 200B amplifier, a Digidata 1200 analog to digital converter and PClamp8 software. The bath temperature was monitored with a thermistor and an inline heater was used to heat the Tyrode’s solution. Both the thermistor and the heater were controlled by a Warner Instruments’ TC-324B bath temperature controller. The bath solution was cooled by an inline ethanol/ice bath. Following establishment of the whole cell voltage clamp configuration the series resistance was compensated by 50 to 60% and cell capacitance was compensated using the settings on the amplifier.

Voltage protocols for voltage gated sodium channels, voltage gated potassium channels and hyperpolarization-activated cyclic nucleotide-gated channels (HCN) were as previously described [9, 11–13]. Briefly, sodium channels were activated by hyperpolarizing the cell membrane to -100mV for 500ms from a holding current of -60mV. The potential was then stepped in 10mV increments from -60mV to 10mV for 2ms. To activate voltage gated potassium channels the cells were hyperpolarized to -100mV for 500ms and then stepped from -60 to 40mV in 20 mV increments for 200ms. The HCN channels were activated by hyperpolarizing the cell membrane stepwise in 10mV increments from -60mV to -120mV for 500ms.

Ramp currents were collected by stepping the voltage to -100mV for 1s followed by a linear voltage ramp from -100mV to +100mV over 4s. Ramp currents obtained in the absence of menthol were subtracted from currents obtained in the presence of 500μM menthol.

### Behavior

Hairless SKH1 mice (Charles Rivers, Wilmington, MA, 20 – 35g) were tested in Orofacial Pain Assessment Devices (OPAD, Stoelting Co., Wood Dale, IL). OPADs utilize a reward/conflict paradigm that requires the mice to place their faces onto temperature programmable Peltier bars in order to obtain a reward solution [14–27]. The reward solution consisted of sweetened condensed milk diluted 1:2 with water. The animals’ food was removed from their home cages 12 to 15 hours prior to testing, but they received water *ad libitum* throughout the fasting period. The mice were trained over a two-week period at three sessions per week with the Peltier bars set at 33°C. The OPADs were then programed to test the animals at 33°, 18° and 10°C during a session. The animals were tested three times a week for up to five weeks.

### Modeling membrane properties

Membrane properties were modeled using the modified Nernst equation:

$$V_{m(t)}=\frac{RT}{zF}∙\frac{P_{h(t-n)}{\left[{Na}^{+}\right]}_{out}+P_{k(t-1)}\;{\left[K^{+}\right]}_{out}}{P_{h(t-n)}{\left[{Na}^{+}\right]}_{in}+P_{k(t-1)}{\left[K^{+}\right]}_{in}}$$

where P_h_ and P_k_ are the relative permeability of I_h_ and I_k_ respectively. The relative difference in the time constants (τ) for I_h_ and I_k_ was compensated for by using n. Ion permeability was estimated using lookup tables generated from non-linear curve fitting of conductances produced from the ion channels’ current/voltage relationships for male and female neurons at 30° and 18°C.

### Statistics

Data collected with PClamp8 were analyzed with Clampfit (Axon Instruments) and then transferred to PRISM6 (GraphPad Software inc., La Jolla, CA) for statistical analysis. Data on the OPADs was collected using AnyMaze (Stoelting Co., Wood Dale, IL) and was also transferred to PRISM6. All data were subjected to Robust regression and Outlier removal (Rout) analysis in PRISM6 with Q = 1% [28]. However, no outliers were identified in the data. Data were then subjected to t-tests, One-Way ANOVAs, Two-Way ANOVAs or Two-Way Repeated Measures ANOVAs as appropriate. Post hoc tests included Tukey’s test, Bonferroni’s test and Sidak’s multiple comparisons test. Alpha was set to 0.05 for all experiments. All data are expressed as means ± SEM.

## Results

### Anatomy of TRPM8 TRG neurons

**Fig 1:**
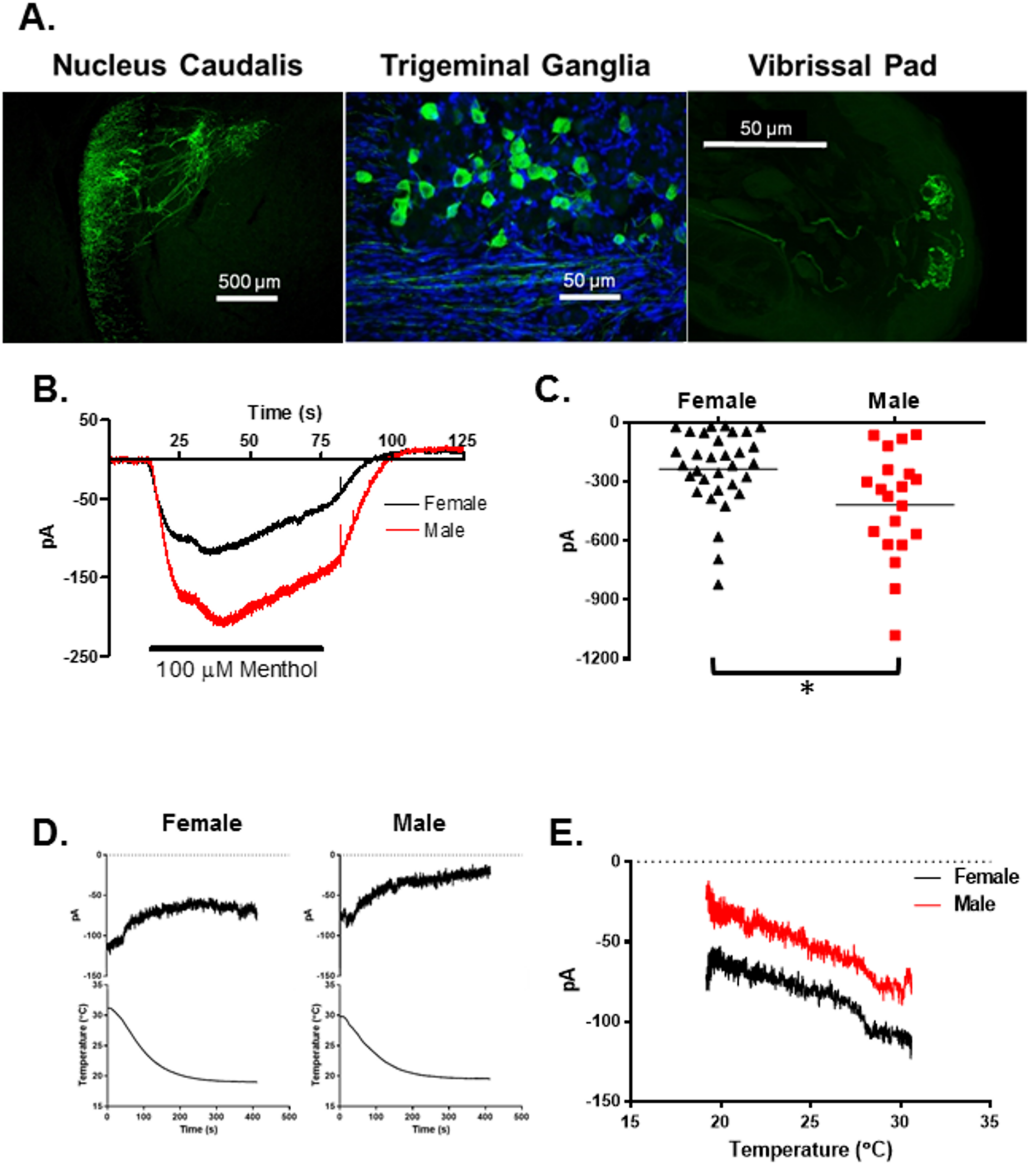
TRPM8 expression and function in TRG neurons. (A) Distribution of TRPM8 in the trigeminal nucleus caudalis, trigeminal ganglia and skin. Nuclei in the ganglia where stained with DAPI. (B) Averaged current traces during the bath application of menthol (100μM) in male and female TRPM8 expressing TRG neurons. Bath temperature was 22°C. N = 20 male neurons from 4 mice, N = 32 female neurons from 7 mice. (C) Comparison of the peak currents from B. Asterisk indicates p = 0.0066, t-test. (D) Averaged holding currents (holding potential -60mV) in female and male TRPM8 TRG neurons during bath cooling. N = 14 female neurons from 3 mice, N = 11 male neurons from 3 mice. (E) Averaged holding currents (holding potential -60mV) versus temperature relationships for the data in D.

Heterozygote mice bred from pairing TRPM8^tm1Apat^/J knockout mice with C57BL/6 mice (TRPM8^EGFP-/+^, N = 3 male and 3 female mice) were used for immunohistochemical evaluation of TRPM8 neurons. These heterozygote mice express EGFP in TRPM8 expressing neurons. As demonstrated in Fig 1A the EGFP labeled TRPM8 neurons are easily identified within the trigeminal ganglia and their axons extend into the trigeminal nucleus caudalis via the spinal tract of V. The fibers synapse primarily in superficial layers of the nucleus with some fibers extending farther into the nucleus. The peripheral terminals of the TRPM8 neurons extend to the skin and end in glomerular like structures near the surface. The glomeruli were approximately 20μm in diameter. There were no significant differences in the number or size of the TRPM8 neurons in the TRGs of male and female mice, nor were any anatomical differences noticed.

### Ion channel properties of male and female TRPM8 TRG neurons

The trigeminal ganglia were removed from the mice, dissociated and plated as described in the methods section. The EGFP labeled neurons were identified with an inverted microscope equipped with fluorescence optics and then the cells were whole cell voltage clamped. To examine the function of TRPM8 in the neurons the agonist menthol (100μM) was added to the bath solution at 22°C, which produced inward currents in the cells (Fig 1B and 1C). The menthol induced currents were significantly larger in TRPM8 neurons from male mice as compared to female mice (N = 20 male neurons, N = 32 female neurons, p = 0.0066, t-test). Cooling the neurons from 30° to 18°C slightly reduced the holding currents (Fig 1D) and female neurons had slightly larger inward holding currents across all temperatures than males (Fig 1E) (N = 14 female neurons, N = 11 male neurons). Cooling the neurons did not appear to activate TRPM8 currents.

**Fig 2:**
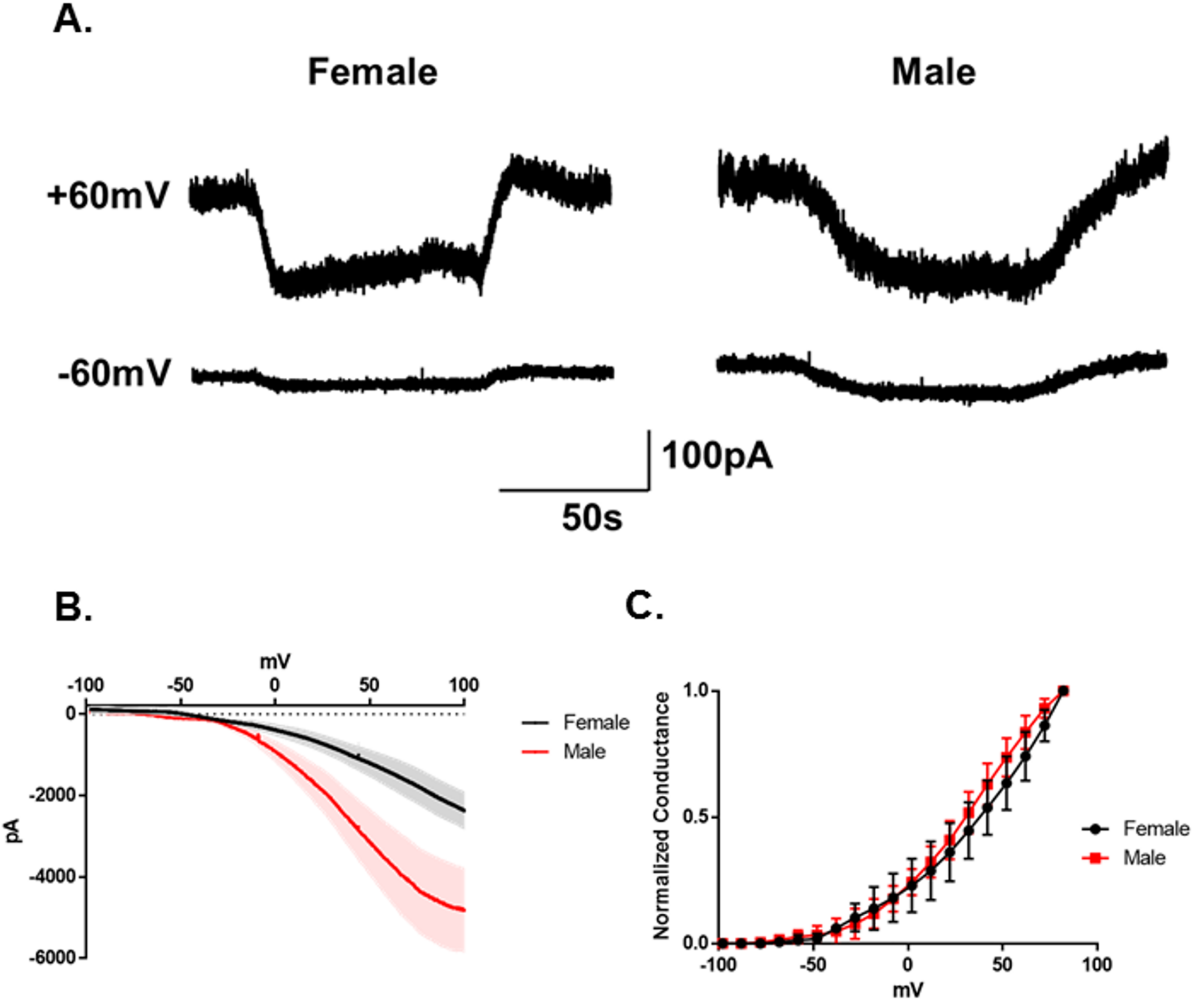
Voltage regulation of TRPM8 in TRG neurons. (A) Representative menthol (500μM) evoked currents in female and male TRPM8 expressing TRG neurons at +60mV and -60mV. (B) Averaged menthol evoked ramp currents for female and male mice. The shaded areas are SEM. (C) Normalized conductances versus voltage. (p = 0.38 2-Way Repeated Measures ANOVA, N = 3 female neurons from 2 mice, N = 6 male neurons from 2 mice).

Previous studies indicated that TRPM8 currents were voltage regulated [5, 29–40]. As illustrated in Fig 2A, bath application of menthol (500μM) produces substantially larger currents at +60mV than at -60mV (N = 3 neurons from 2 female mice, N = 6 neurons from 2 male mice). Voltage ramp analysis of the menthol evoked currents in these neurons confirmed the voltage sensitivity of the channels and further confirmed that the menthol evoked currents from male neurons were significantly larger than the currents from female neurons (Fig 2B). However, the sex of the animals did not alter the kinetics of the channels (Fig 2C, p = 0.38 2-Way Repeated Measures ANOVA).

**Fig 3:**
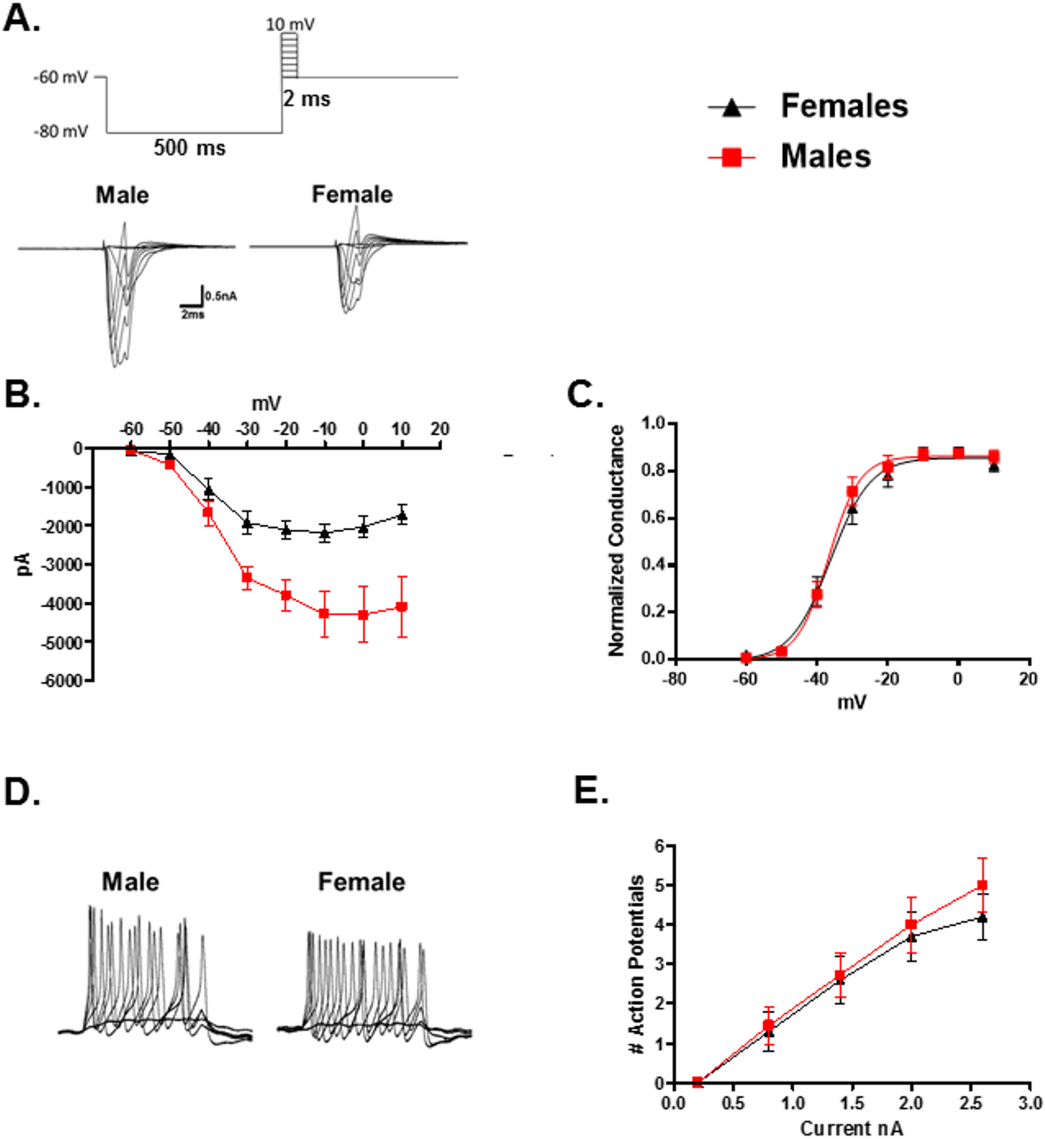
Comparison of voltage gated sodium channels in male and female TRPM8 expressing TRG neurons. (A) Voltage protocol used to evoke currents in voltage activated sodium channels. Traces are representative currents for male and female TRPM8 expressing TRG neurons. (B) Current/voltage relationships for male and female voltage gated sodium channels in TRPM8 expressing TRG neurons. N = 29 male neurons from 5 mice, N = 39 female neurons from 9 mice, p = 0.0005, 2-Way Repeated Measures ANOVA. (C) Normalized conductance versus voltage plot for voltage gated sodium channels in TRPM8 expressing TRG neurons. (D) Evoked action potentials in representative current clamped TRPM8 expressing TRG neurons from a male and female. (E) Relationship between injected current and the number of evoked action potentials. N = 11 male neurons from 4 mice, N = 10 female neurons from 2 mice, p = 0.67, 2-Way Repeated Measures ANOVA.

Additionally, it was found that male TRPM8 neurons had larger voltage gated sodium currents than females (Fig 3A and 3B, N = 29 male neurons, N = 39 female neurons, p = 0.0005, 2-Way Repeated Measures ANOVA). However, activation kinetics for male and female sodium currents were nearly identical (Fig 3C, p = 0.65, 2-Way Repeated Measures ANOVA). Injecting depolarizing current for 100ms under current clamp conditions induced similar numbers of action potentials in both male and female TRPM8 neurons (Fig 3D and 3E, N = 11 male neurons, N = 10 female neurons, p = 0.67, 2-Way Repeated Measures ANOVA). The peak voltage of the action potentials also did not differ between males (27.3 ± 2.1mV) and females (29.3 ± 1.3mV) (N = 20 male neurons from 4 mice, N = 10 female neurons from 2 mice, p = 0.43, t-test). There was also no difference in resting membrane potential at room temperature (22°C) between the sexes (N = 20 male neurons from 4 mice: R_m_ = -54.9 ± 1.2mV; N = 10 female neurons from 2 mice: R_m_ = -54.2 ± 1.0mV, p = 0.68, t-test).

**Fig 4:**
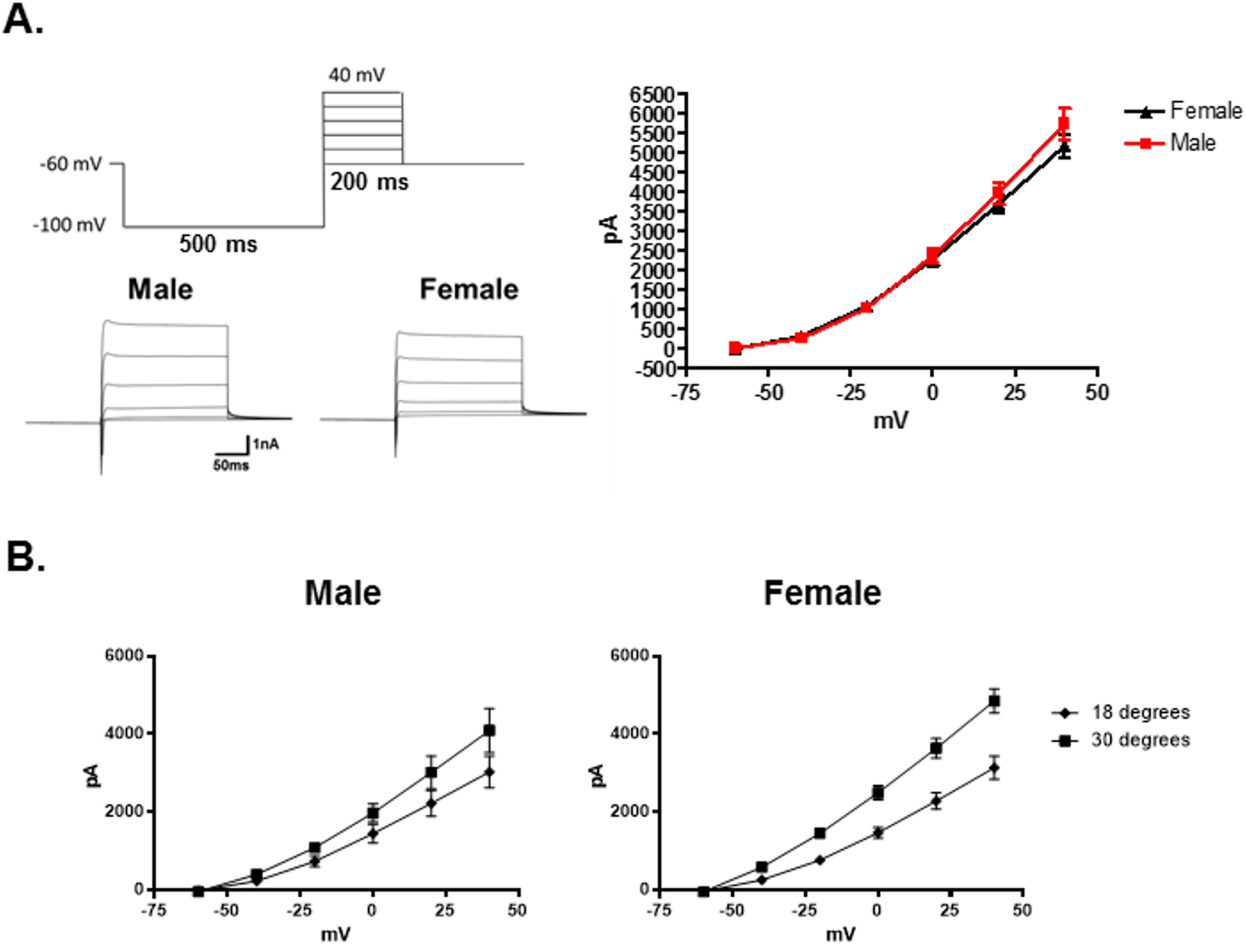
Comparison of voltage gated potassium channels in male and female TRPM8 expressing TRG neurons. (A) Voltage protocol for activating voltage gated potassium channels in TRPM8 expressing TRG neurons. Representative currents for a male and female neuron are presented below the protocol. The graph illustrates that at 22°C the current amplitude between male and female neurons did not differ. N = 29 male neurons from 5 mice, N = 46 female neurons from 9 mice, p = 0.22, 2-Way Repeated Measures ANOVA. (B) The voltage activated potassium channel currents (I_k_) from female TRPM8 expressing neurons are significantly suppressed at 18°C when compared to 30°, whereas male currents are not. Females 30°: N = 23 neurons; 18°: N = 13 neurons 5 mice, p = 0.0002, 2-Way Repeated Measures ANOVA. Males 30°: N = 12 neurons; 18°: N = 11 neurons, 3 mice, p = 0.12, 2-Way Repeated Measures ANOVA.

Additionally, there were no significant differences between the sexes in outward currents produced by voltage dependent potassium channels (I_k_) at 22°C (Fig 4A, N = 29 male neurons, N = 46 female neurons, p = 0.22, 2-Way Repeated Measures ANOVA).

To examine temperature sensitivity, the EGFP labeled neurons were voltage clamped and the temperature of the bath was set at 30°C. The temperature was then ramped to 18°C over the course of 30 seconds. Voltage gated sodium currents were reduced by cool temperatures, but there was no significant difference between neurons from males and females in their temperature response profile. On the other hand, female neurons exhibited a significant difference in I_k_ when comparing amplitudes at 30°C and 18°C (Fig 4B, 30°: N = 24 neurons; 18°: N = 12 neurons, p = 0.0002, 2-Way Repeated Measures ANOVA), whereas male neurons did not demonstrate significant temperature sensitivity in these currents (30°: N = 12 neurons; 18°: N = 11 neurons, p = 0.12, 2-Way Repeated Measures ANOVA). Likewise, the activation constant (τ) for I_k_ at 40mV was significantly increased by the lower temperature in the female neurons, but not in the male neurons (One-Way ANOVA, p < 0.0001; Female (5 mice): 30°C: N = 24 neurons, τ = 0.25 ± 0.02ms; 18°C: N = 12 neurons, τ = 1.34 ± 0.32ms, Tukey test p < 0.05; Male (3 mice): 30°C: N = 12 neurons, τ = 0.35 ± 0.09ms; 18°C: N = 11 neurons, τ = 0.88 ± 0.18ms, Tukey test p > 0.05).

**Fig 5:**
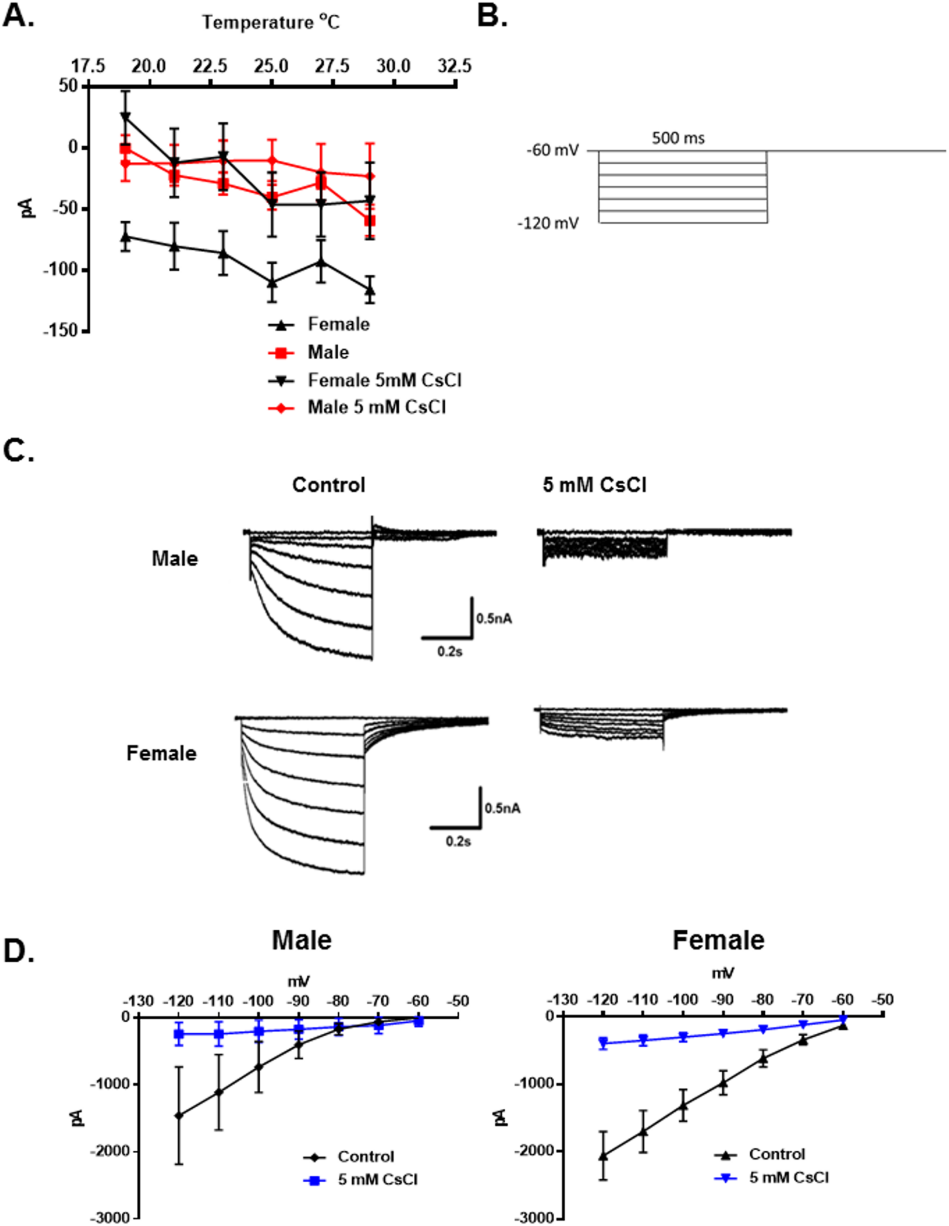
Effect of 18°C on holding currents mediated by hyperpolarization-activated cyclic nucleotide-gated channels (HCN) in male and female TRPM8 expressing TRG neurons. (A) Neurons from female mice had larger inward holding currents at all temperatures tested (holding potential -60mV) N = 24 male neurons from 3 mice, N = 30 female neurons from 6 mice, p < 0.0001, 2-Way Repeated Measures ANOVA. Cesium (5mM) suppressed the female current, N = 7 neurons from 2 mice. Cesium did not influence the male current, N = 6 neurons from 2 mice. (B) Voltage protocol to evoke HCN currents (I_h_). (C) Cesium suppressed I_h_ in both female and male neurons. Traces represent the averaged currents. N = 5 female neurons from 1 mouse, p = 0.004, 2-Way Repeated Measures ANOVA; N = 7 male neurons from 2 mice, p = 0.005, 2-Way Repeated Measures ANOVA.

**Fig 6:**
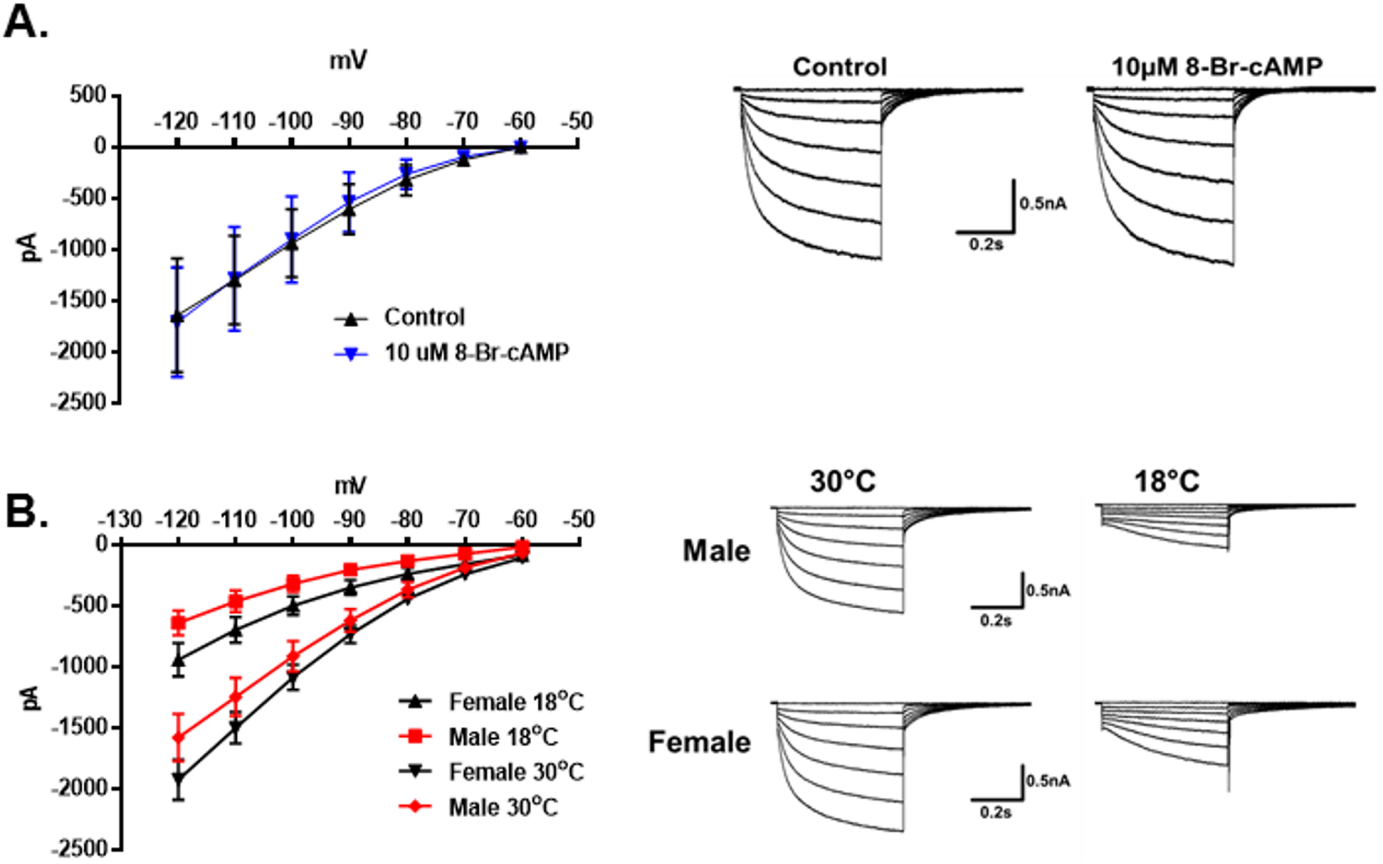
Cyclic nucleotide and temperature dependence of HCN currents in TRPM8 expressing neurons. (A) 8-bromo-cAMP (10μM) had no effect on the I_h_ in female TRPM8 expressing TRG neurons. N = 5 female neurons from 2 mice, p = 0.91, 2-Way Repeated Measures ANOVA. The traces to the right are the average of all the traces in the absence and presence of 8-bromo-cAMP. (B) Effect of temperature on I_h_ in male and female TRPM8 expressing TRG neurons. Both female and male neuron I_h_ were suppressed by 18°C. Female (5 mice) 30°: N = 25 neurons; 18°: N = 13 neurons p < 0.0001, 2-Way Repeated Measures ANOVA; Male (3 mice) 30°: N = 12 neurons; 18°: N = 11 neurons, p = 0.0001, 2-Way Repeated Measures ANOVA. Female I_h_ was larger than male I_h_ at both 18° and 30°C. Male vs female 30°C: p = 0.013, 2-Way Repeated Measures ANOVA. Male vs female 18°C: p < 0.0001, 2-Way Repeated Measures ANOVA. Traces to the right are averages of the currents at 18° and 30°C for male and female TRPM8 expressing TRG neurons.

The protein TRPM8 is a temperature sensitive ion channel that is reported to be activated at temperatures below 22°C [3, 7]. However, in contrast to the previous studies there was no apparent activation of TRPM8 currents during the temperature ramps (30° to 18°C). Instead it was found that the inward holding current (at a holding potential of -60mV) was significantly larger for females than males. The holding current decreased as the temperature was reduced in both sexes. In male neurons this current approached 0pA at 18°C. In female neurons it approached -50pA (Fig 5A, N = 24 male neurons, N = 30 female neurons, p < 0.0001, 2-Way Repeated Measures ANOVA) (also see Fig 1D and 1E). It was hypothesized that the current was mediated by hyperpolarization-activated cyclic nucleotide-gated channels (HCN). These channels are blocked by external cesium ions [41–46]. As demonstrated in Fig 5A, 5mM cesium suppressed the inward current in the female neurons to match the holding current in males (N = 7 female neurons). Cesium did not significantly influence the male holding current (N = 6 male neurons). To confirm the identity of the inward current the membrane potential was stepped from -60mV to -120mV in 10mV increments for 500ms to activate HCN channels (Fig 5B). In both female and male TRPM8 expressing neurons the HCN currents (I_h_) were almost completely blocked by 5mM extracellular cesium (Fig 5C and 5D, N = 5 female neurons, p = 0.004, 2-Way Repeated Measures ANOVA, N = 7 male neurons, p = 0.005, 2-Way Repeated Measures ANOVA). However, although many HCN channels are activated by cyclic nucleotides [42], I_h_ in the female TRPM8 TRG neurons was not significantly influenced by 8-bromo-cAMP (10μM) (Fig 6A, N = 5 female neurons, p = 0.91, 2-Way Repeated Measures ANOVA). As suggested by the temperature dependence of the holding current, I_h_ in both sexes was significantly reduced at 18°C compared with 30°C (Fig 6B, Female 30°: N = 25 neurons; 18°: N = 13 neurons, p < 0.0001, 2-Way Repeated Measures ANOVA; Male 30°: N = 12 neurons; 18°: N = 11 neurons, p = 0.0001, 2-Way Repeated Measures ANOVA). Additionally, I_h_ was larger in females at both temperatures (Fig 6B, male vs female 30°C, p = 0.013, 2-Way Repeated Measures ANOVA; male vs female 18°C, p < 0.0001, 2-Way Repeated Measures ANOVA). The τ for activation of I_h_ at -120mV was not significantly different between male and female neurons nor did temperature significantly affect τ (One-Way ANOVA, p = 0.10; Male 30°C: N = 11 neurons, τ = 263.8 ± 32.3ms; 18°C, N = 10 neurons, τ = 444.0 ± 163.8ms; Female 30°C: N = 19 neurons, τ = 209.2 ± 13.6ms; 18°C, N = 11 neurons, τ = 372.9 ± 70.3ms).

### Behavioral analysis of male and female responses to neutral, cool and cold temperatures

**Fig 7:**
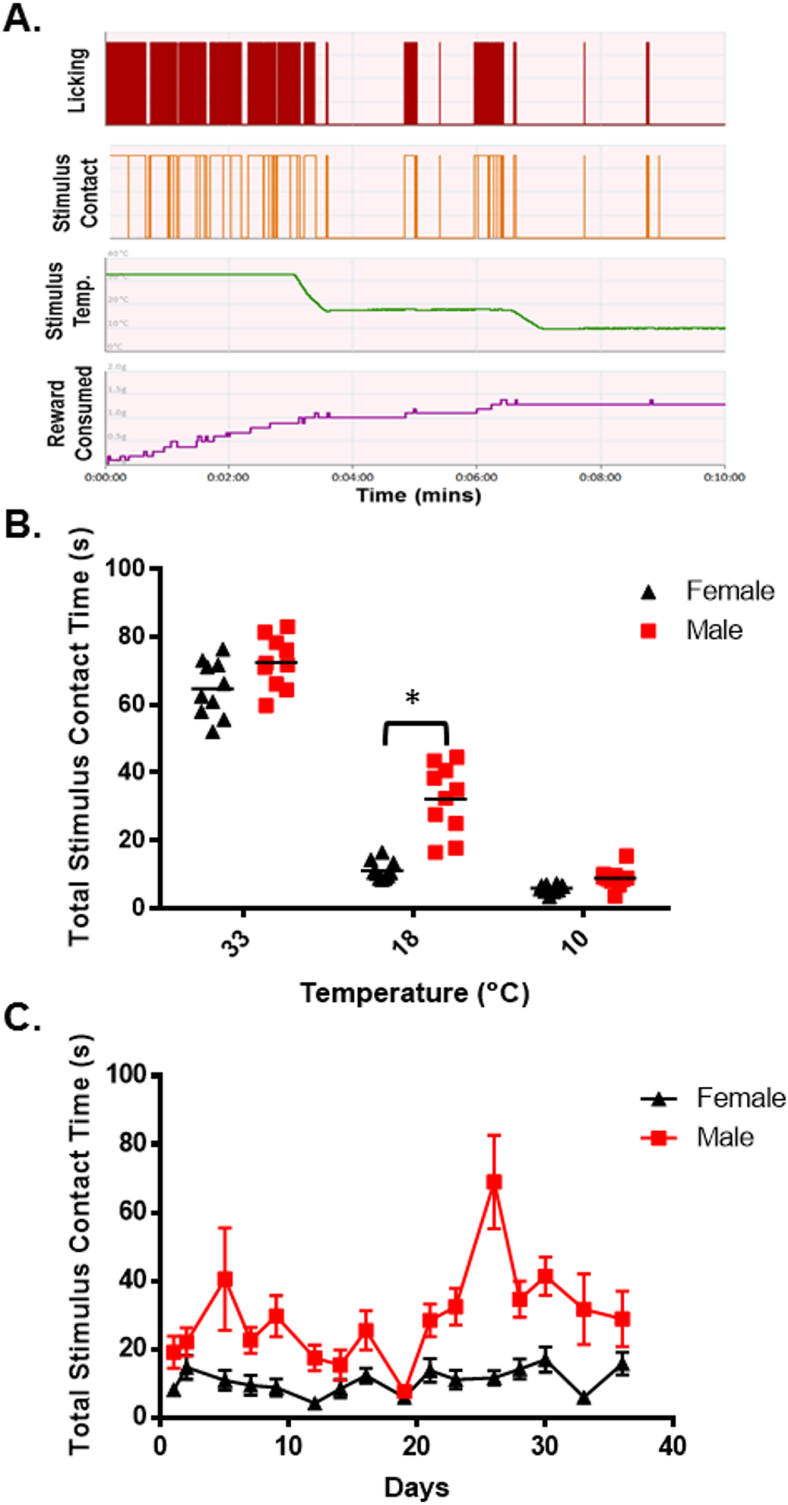
Sex differences in behavioral responses to thermal stimuli. (A) Representative Data collected on an Orofacial Pain Assessment Device (OPAD) from a male SKH1 mouse. (B) Stimulus contact time for male and female mice when the Peltiers were set to 33°, 18°, and 10°C. The data collected from 16 trials were averaged for each mouse and then plotted as the mean ± SEM. Asterisk indicates p = 0.0002, 2-Way ANOVA, Bonferroni’s test. N = 10 male and 10 female mice. (C) Behavioral responses to 18°C on the OPADs throughout the time course of the experiment. N = 10 male and 10 female mice, p < 0.0001 Repeated Measures 2-way ANOVA.

Neurons that express the temperature sensitive ion channel TRPM8 were reported to detect cool temperatures [3, 7]. Since male and female TRPM8 expressing neurons demonstrated distinct differences in their responses to the TRPM8 agonist menthol and differences in voltage gated sodium and potassium currents, as well as HCN mediated currents, it was hypothesized that male and female mice would respond differently to cool or cold temperatures in behavioral assays. To test this hypothesis male and female hairless SKH1 mice were tested in an operant orofacial pain assessment device (OPAD) at 33°C, 18°C and 10°C [14–19, 21, 25, 47, 48] (Fig 7A). Hairless mice were used to eliminate the insulating effect of the animals’ fur in the assay. Males and females were also tested in separate sets of OPADs to reduce sex odor related distractions during the assay. As Fig 7B demonstrates male and female mice contacted the 33°C and 10°C stimulus for a similar amount of time during the assay, but at 18°C male mice contacted the stimulus for more than twice as long as female mice (N = 10 male and 10 female mice, p = 0.0002, 2-Way ANOVA, Bonferroni’s test). These findings suggest that the female mice were more sensitive to the cool temperature. Fig 7C illustrates that this sex difference in 18°C stimulus contact time was maintained throughout the 36 days that the animals were tested.

## Discussion

Human females frequently report discomfort in cool environments, while their male counterparts find the same temperature comfortable or even a bit warm [1]. These data suggest that males and females differ in processing environmental temperature information. It was previously demonstrated that cool temperatures are detected by neurons using the temperature sensitive protein TRPM8 [3–8, 15, 49–51]. The primary afferent neurons that express this protein are presumed to detect environmental cool or cold temperatures at the skin surface and then transmit the signal centrally. The differences between the sexes in the perception of cool temperatures could thus be manifested in the primary afferents fibers if they differed in the expression or function of TRPM8, or some other ion channel that resulted in a difference in the frequency or pattern of their action potentials in response to low temperatures. Alternatively, the cool sensing primary afferent fibers may not differ and the female sensitivity arises from central processing of the incoming signal. In this study, we examined TRPM8 expressing neurons in male and female mouse trigeminal ganglia to determine if there was a difference in their TRPM8 channels, voltage gated channels or temperature sensitivity. We found that male TRPM8 TRG neurons had larger menthol induced inward currents than female neurons (Figs 1B, 1C and 2B). This finding would suggest that male mice would be more sensitive to cool temperatures than female mice. However, behavioral experiments demonstrated greater sensitivity to 18°C in female mice (Fig 7B and 7C). And, interestingly, cooling the neurons to 18°C did not visibly activate TRPM8 in these TRG neurons (see Figs 1D, 1E and 5A). This finding contrasts with previous reports [3–5, 7, 8, 49]. However, TRPM8 is voltage and cold gated [5, 31–40]. We confirmed the voltage dependence of TRPM8 (Fig 2A, 2B and 2C). At voltages below 0mV and at cool temperatures the current is significantly depressed. The reduced TRPM8 current at -60mV coupled with the suppression of I_h_ by the cool temperatures likely masked any activation of the TRPM8 channels by cooling. These findings also indicate that differences in TRPM8 are not likely to produce the sex differences in response to cool temperatures measured using the OPAD assay.

Analysis of voltage gated currents found that sodium currents were substantially larger in male neurons than female neurons (Fig 3A and 3B). The larger currents did not translate into a difference in the number of action potentials generated by depolarizing the cells (Fig 3D and 3E), nor did it translate into larger action potentials, a difference in response to cool temperatures, or a difference in activation kinetics (Fig 3C). Thus, it is not clear that the difference in sodium current amplitude has any functional significance in defining the sex difference in response to cool temperatures.

In examining the effects of cooling on holding currents in the TRPM8 expressing TRG neurons it was found that females had larger inward holding currents at -60mV at all temperatures tested (Figs 1D, 1E and 5A). The holding currents in both sexes decreased linearly with the temperature and at 18°C the current in male neurons approached zero, whereas the inward current in females remained significantly larger. We hypothesized that the inward holding current was mediated by HCN channels, which are blocked by extracellular cesium ions [42]. Cesium blocked the holding current in female neurons confirming the hypothesis. We further evaluated I_h_ and found that at both 30°C and 18°C I_h_ was larger in female neurons than in male neurons (Fig 6B). The current was also blocked by extracellular cesium (Fig 5C and 5D), was insensitive to 8-bromo-cAMP (Fig 6A), and had fast activation kinetics. There are four HCN channels that are activated to different degrees by cyclic nucleotides and have distinct kinetics. HCN1 is the most resistant to activation by cyclic nucleotides and is fast activating [42, 44], thus the data suggest that I_h_ and the holding current are mediated by HCN1 channels. Thus, the data suggest that HCN1 channels participate in producing the sex difference in cool temperature sensitivity.

**Fig 8:**
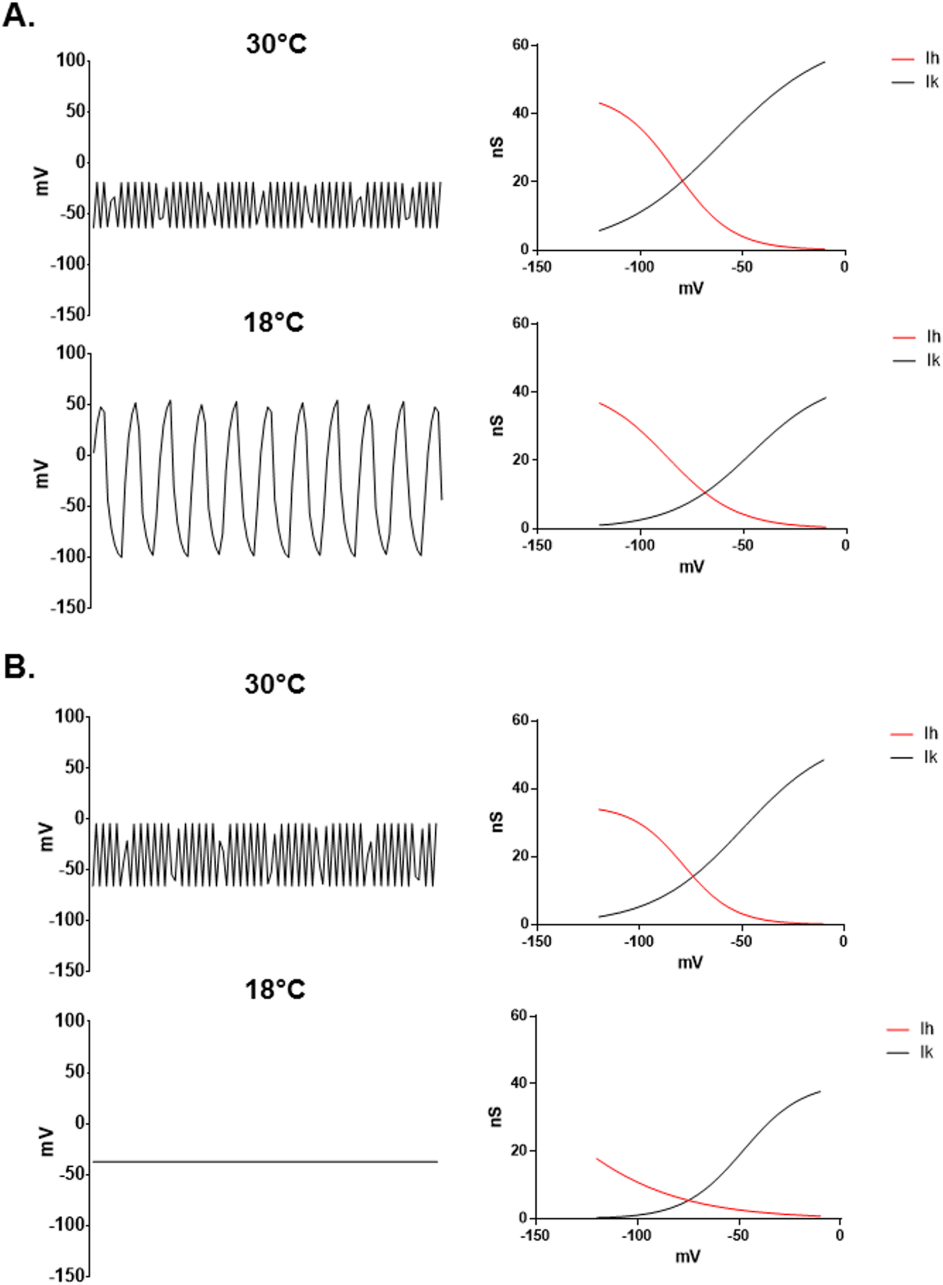
Modeling membrane potential oscillations in male and female TRPM8 TRG neurons at 30° and 18°C. The modified Nernst equation presented in the methods was used to simulate membrane potentials. The value n was set to 13 in the model for this figure. However, similar results were obtained with all values of n > 5. (A) In female neurons the cooler temperature reduces both I_k_ and I_h_ resulting in a lower frequency and larger fluctuations in membrane potential. The graphs to the right of the traces are plots of the conductances used in the lookup table for the model. (B) In male neurons I_h_ is reduced at cool temperatures while I_k_ remains unaffected, resulting in a net suppression of membrane oscillations. The graphs to the right of the traces are plots of the conductances used in the lookup table for the model.

The hypothesis that HCN1 mediates the sex difference in TRPM8 TRG neuronal responses to cool temperatures is consistent with the findings of Orio and colleagues who demonstrated that HCN1 mediates oscillations in the membrane potential of cold sensitive TRG neurons [43, 52]. Blocking or knocking out HCN1 reduces the sensitivity of mice to cold temperatures. Although they did not examine sex differences, Orio et al. found that these neurons fired spontaneous bursts of action potentials. Interestingly, the interburst interval increased as the temperature was reduced, while at the same time the number of action potentials per burst increased. This pattern resulted not only in a new pattern of firing, but an actual increase in the overall action potential firing rate [43, 52]. Modeling membrane dynamics using the I_h_ and I_k_ currents found in this project demonstrates that both male and female neurons would be expected to have relatively unstable membrane potentials at 30°C (Fig 8). The model did not account for currents other than I_h_ and I_k_, but was intended to illustrate the dynamic interaction of these two currents as they respond to different temperatures. As predicted from Orio et al.’s work [43, 52], however, female neurons demonstrate large oscillations in membrane potential with a reduced frequency at 18°C (Fig 8A). The membrane potential of male neurons, on the other hand, becomes much more stable at 18°C than it was at 30°C (Fig 8B).

HCN channels are responsible for pacemaker activity in both heart cells and neurons [53–55]. In this study, through a pacemaker like function, I_h_ mediated encoding of the cool signal within the TRPM8 expressing neurons of females. The current allows a slow depolarization of the membrane potential until a burst of action potentials occurs, which then activates I_k_ to repolarize the cell. Interestingly, our finding that the female I_k_ in TRPM8 TRG neurons was significantly reduced by cool temperatures, along with an increase in its activation constant (τ), would favor bursting behavior (Fig 8A). The slow onset and reduced amplitude of I_k_ at cool temperatures allows the cell to depolarize more before I_k_ returns the membrane potential below the action potential threshold, which in turn produces larger bursts of action potentials. As a result of the cooling effect on both I_h_ and I_k_, female neurons preserve a relative balance between I_h_ and I_k_ (I_k(40mV)_/I_h(-120mV)_, 30°C: -2.630 ± 0.286, 18°C: -3.584 ± 0.621), retaining an unstable membrane potential at cool temperatures. Since TRPM8 is both voltage and temperature dependent, making the channel relatively inactive at resting membrane potentials and 18°C [5, 6, 30–40], the model suggests that the unstable dynamics of the female membrane potential at 18°C could recruit TRPM8 to participate in detecting the cool temperature. In this scenario, the membrane potential is raised above 0mV by I_h_, which then allows TRPM8 to be fully activated by the cool temperature. On the other hand, I_k_ in male neurons was not significantly altered by cool temperatures (Fig 4B), while I_h_ was reduced by cooling (Fig 6B). Thus, at cool temperatures male neurons become dominated by I_k_ (I_k(40mV)_/I_h(-120mV)_, 30°C: -2.690 ± 0.490, 18°C: -4.912 ± 0.937), which suppresses burst firing, as was demonstrated by the model. The stable membrane potential of the male neurons at cool temperatures prevents the recruitment of TRPM8. These data suggest that in vivo the female neurons may exhibit profound bursting behavior at 18°C as described by Orio et al. [43, 52], while the male neurons may actually lose their bursting characteristics as the inhibitory current I_k_ dominates the membrane potential. Thus, our data indicate that at 18°C I_h_ and I_k_ combine to mediate distinct patterns of neuronal activity in male and female TRPM8 TRG neurons that may underlie the sex difference in sensitivity to 18°C.

Sex differences in sensitivity to cool temperatures have been reported both anecdotally and experimentally in humans for many years. Our findings indicate that these same sex differences exist in mice and the data suggest that the mechanism for these differences may involve HCN1 channels and voltage gated potassium channels in TRPM8 expressing primary afferent neurons. This conclusion is supported by the work of Orio et al. [43, 52] who utilized HCN knockout mice to verify the role of HCN channels in cool sensation. However, HCN channels are responsible for pacemaker activity in the heart and participate in learning and memory, as well as other neuronal functions [42, 44–46, 56]. Thus, any conclusions based on behavioral responses examined in HCN knockout animals or animals that are treated with HCN blockers must be tempered by the fact that several bodily functions are compromised by the inhibited HCN channels. In experiments designed to test the role of HCN channels in the sex differences in temperature sensitivity we utilized the HCN blocker ZD7288 (5mg/kg)[43, 44, 52, 54] in male and female mice in OPADs. The agent not only suppressed the difference between the sexes at 18°C, but it drastically reduced overall performance in the assay, suggesting that the animals were not healthy after treatment (S1 Fig). Since this dose is lower than is typically used for behavioral studies, the reduced performance of the animals in the OPAD assay indicates that the results were compromised and that verifying the role of HCN channels in behavioral assays will require TRPM8 neuron specific inactivation of the channels. Similarly, voltage gated potassium channels are critical for a number of functions and, thus, attempting to manipulate them in vivo to examine their role in temperature sensitivity will lead to confounded results.

Beyond the temperature differences in sensory function, the current findings also suggest that males and females may have many other differences in primary afferent ion channels that mediate other sensory functions. These differences may impart particular vulnerability to chronic pain conditions that have a higher prevalence in one sex over the other. Further studies are needed to elucidate these differences in male and female primary afferent neurons.

## Supporting information

S1 Fig Effect of blocking HCN channels in vivo on OPAD performance at 18°C. Ten male and ten female SKH1 mice were trained in the OPAD assay as described in the methods and then tested at 18°C. The male and female mice were tested in separate OPAD devices to limit interference in the assay from sex odors. Two days following control testing the same animals were given 5mg/kg ZD7288 (ip. In saline) and then tested 30 minutes later in the OPADs at 18°C. Asterisk indicates p < 0.05, 2-Way ANOVA followed by Sidak’s Multiple Comparison’s test, Control Male versus Control Female. It was also noted that the animals were lethargic, suggesting that cardiovascular function was compromised by the agent.
(DOCX)
