# Supplementary material for: Sex differences in mouse Transient Receptor Potential Cation Channel, Subfamily M, Member 8 expressing trigeminal ganglion neurons
Source: PLoS One. 2017 May 4;12(5):e0176753. doi: 10.1371/journal.pone.0176753 (PMC5417611; doi:10.1371/journal.pone.0176753)
Supplement: S1 Fig — Ten male and ten female SKH1 mice were trained in the OPAD assay as described in the methods and then tested at 18°C. The male and female mice were tested in separate OPAD devices to limit interference in the assay from sex odors. Two days following control testing the same animals were given 5mg/kg ZD7288 (ip. In saline) and then tested 30 minutes later in the OPADs at 18°C. Asterisk indicates p < 0.05, 2-Way ANOVA followed by Sidak’s Multiple Comparison’s test, Control Male versus Control Female. It was also noted that the animals were lethargic, suggesting that cardiovascular function was compromised by the agent. (DOCX) [file pone.0176753.s001.docx]

Fig. S1- Effect of blocking HCN channels in vivo on OPAD performance at 18°C. Ten male and ten female SKH1 mice were trained in the OPAD assay as described in the methods and then tested at 18°C. The male and female mice were tested in separate OPAD devices to limit interference in the assay from sex odors. Two days following control testing the same animals were given 5mg/kg ZD7288 (ip. In saline) and then tested 30 minutes later in the OPADs at 18°C. Asterisk indicates p < 0.05, 2-Way ANOVA followed by Sidak’s Multiple Comparison’s test, Control Male versus Control Female. It was also noted that the animals were lethargic, suggesting that cardiovascular function was compromised by the agent.
